# Chromatin as an old and new anti-cancer target

**DOI:** 10.1016/j.trecan.2024.05.005

**Published:** 2024-06-01

**Authors:** Jacques Neefjes, Katerina Gurova, Jay Sarthy, Gábor Szabó, Steven Henikoff

**Affiliations:** 1– Department of Cell and Chemical Biology and Oncode Institute, LUMC, Einthovenweg 20, 2333 ZC Leiden, the Netherlands; 2– Department of Cell Stress Biology, Roswell Park Cancer Institute, Elm and Carlton Streets, Buffalo, NY, 14263, USA; 3– Department of Pediatrics, University of Washington School of Medicine and Seattle Children’s Research Institute, 1920 Terry Ave, -Seattle, WA 98109, USA; 4– Faculty of Medicine, Department of Biophysics and Cell Biology, University of Debrecen, Debrecen, Egyetem tér 1, 4032 Hungary; 5- Basic Sciences Division, Fred Hutchinson Cancer Center, 1100 Fairview Ave N, Seattle, WA 98109, USA; Howard Hughes Medical Institute, Chevy Chase, MD 20815, USA

**Keywords:** chromatin damage, DNA damage, anthracyclines, cancer, curaxin

## Abstract

Recent genome-wide analyses identified chromatin modifiers as one of the most frequently mutated class of genes across all cancers. However, chemotherapies developed for DNA damage-compromised cancers remain the standard of care for chromatin-deranged malignancies. We address this conundrum by establishing the concept of “chromatin damage”: the non-genetic damage to protein-DNA interactions induced by certain small molecules. We highlight **anthracyclines**, a class of chemotherapeutic agents ubiquitously applied in oncology, as an example of an overlooked chromatin-targeting agent. We discuss our current understanding of this phenomenon and explore emerging chromatin-damaging agents as a basis for further studies to maximize their impact in modern cancer treatment.

## A better look at DNA damage as chemotherapeutic activity

Despite recent developments in targeted therapies, conventional chemotherapy remains the standard of care for many cancers. One reason for the continued use of ‘old chemotherapy’ in cancer is that drug resistance is more difficult to develop as most drugs target DNA. While targeted therapy may be more specific, it can easily result in development of drug resistance through acquiring mutations or activation of parallel signaling pathways. Interpatient and intrapatient heterogeneity of tumors further limits the efficacy of targeted agents [1–3]. As a result, chemotherapy, such as platinum compounds and/or **anthracyclines** (see Glossary), remain cornerstones in cancer treatment despite severe side effects.

**Figure 1,:**
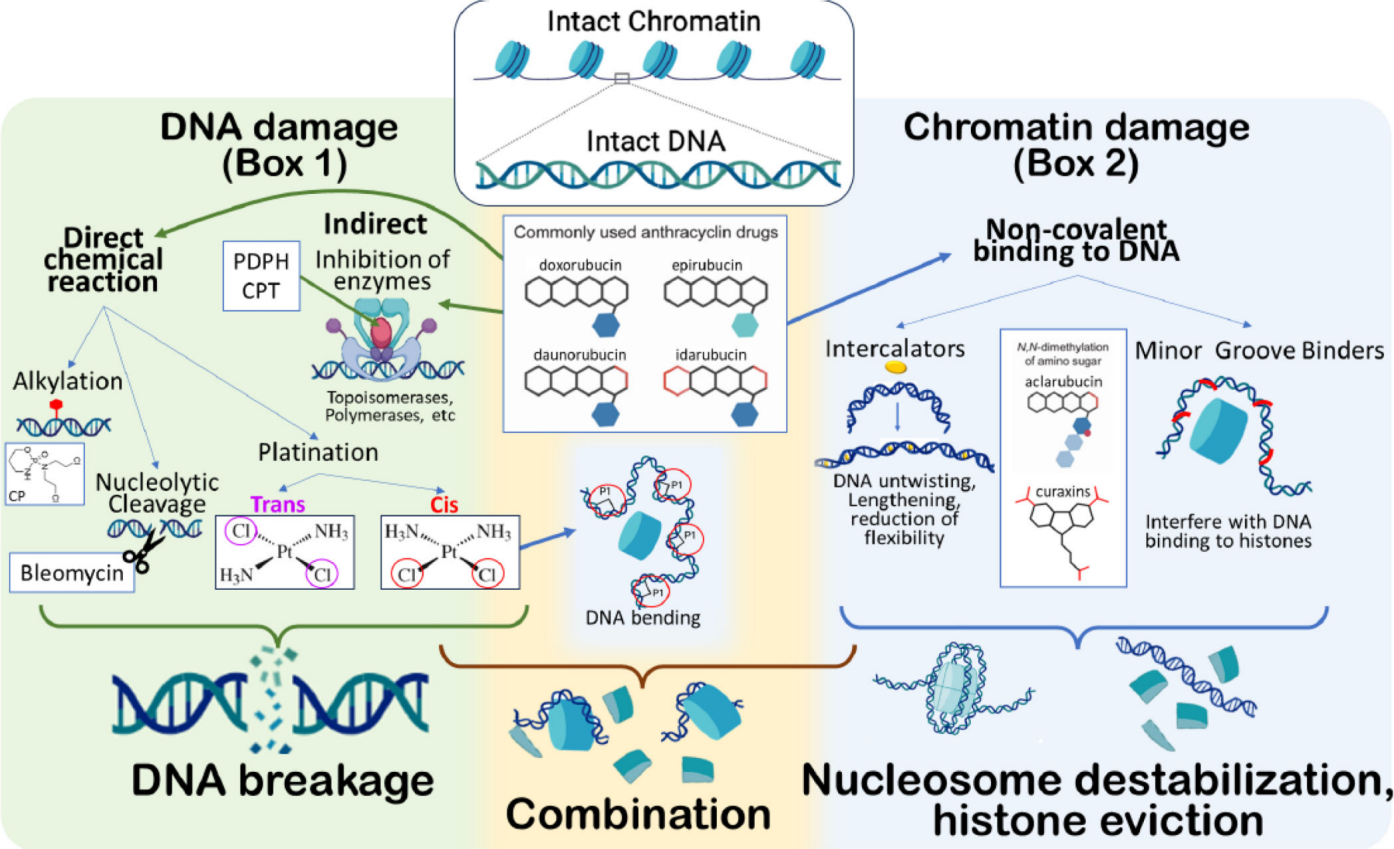
Key Figure. Mechanisms of DNA and chromatin damage caused by small molecules. Two major mechanisms of DNA damage are on the right on the green background (see also details in Box 1). Small molecules can chemically react with DNA via alkylation (e.g., CP -cyclophosphamide), cutting DNA in nuclease-like manner (bleomycin) and interfering with enzymes using DNA as substrate (e.g., binding to a cleavable complex of DNA with topoisomerase I (e.g., CPT – camptothecin) or topoisomerase II (e.g., PDPH – podophyllotoxins). Platinum compounds covalently bind to DNA either in a trans (different DNA strands) or a cis (the same DNA strands) manner. Major mechanisms of chromatin damage are on the left on the blue background. They include non-covalent binding to DNA and interfering with DNA-histone interactions. Some drugs, e.g., most commonly used anthracyclines and cisplatin, cause a combination of DNA and chromatin damage (yellow background). Anthracyclines intercalate DNA and cause histone eviction, but they also inhibit topoisomerases and some of them can cause ROS-dependent DNA oxidation. Cisplatin chemically reacts with DNA and bends DNA causing DNA unwrapping from nucleosome. CP - cyclophosphamide, CPT – camptothecin, PDPH – podophyllotoxins. Created with BioRender.com.

Many chemotherapy drugs directly or indirectly target DNA or DNA-related processes, such as replication, nucleotide synthesis or maintenance of DNA topology (Box 1 and Figure 1, Key Figure). It is believed that tumor cells are more sensitive to agents targeting DNA as mitotic cells cannot handle DNA damage. When cancer cells divide faster than healthy cells, this automatically induces a therapeutical window [4]. This belief persists despite a subset of cancers, such as pediatric malignancies, having low mutational burdens and relatively intact DNA repair pathways [5, 6]. While most childhood cancers can be effectively treated with chemotherapy [7], pediatric cancer patients generally have significantly shortened lifespans and lower quality of life after intense chemotherapy cycles [8].

Chemotherapy is associated with more side effects than targeted therapies. Whether these side effects are related to DNA damage is unclear since different DNA-damaging drugs have different side effects. For example, most DNA damaging drugs cause myelosuppression and gastrointestinal toxicity [9]. While platinum compounds cause peripheral neuropathy, hepatotoxicity and retinopathy [10], widely used anthracyclines cause cardiotoxicity [11]. Moreover, DNA-damaging drugs can be genotoxic and result in therapy-induced secondary tumors and infertility by increasing the DNA mutation rate [12].

The notion of DNA damage being the primary mechanism of anti-cancer activity of all DNA-targeting agents should be reconsidered in view of recent observations. Some anthracycline variants are DNA-binding compounds with anti-cancer activity, but do not cause detectable DNA damage in mammalian cells [13–15]. Moreover, the anti-cancer activity of some of these agents is stronger than that of structurally similar DNA-damaging drugs, and at the same time, they are much less likely to cause long-term side effects [13, 14, 16]. Rather than causing DNA damage, these compounds disturb the packaging of DNA into **chromatin**, leading to chromatin destabilization and histone loss from chromatin at specific loci (Box 2 and Figure 1). Interestingly, the anthracyclines clinically used in the Western world for cancer treatment (doxo-, dauno-, epi- and idarubicin) induce DNA breaks as well as disturb the packaging of DNA and thus may have at least two mechanisms of apoptosis induction.

In this review, we discuss the mechanisms of chromatin destabilization by DNA-binding small molecules, its cytotoxic effects, and therapeutic opportunities. The development of chemotherapies that alter chromatin structure represents a new strategy for improving the efficacy and safety of cancer therapeutics and may define a new target for further drug development.  

## The effect of DNA-binding compounds on chromatin

When DNA-binding compounds were initially identified over five decades ago, it was quickly noticed that they cause DNA unwrapping from nucleosomal cores in cell-free conditions, and loss of histones from chromatin in cells [17–19]. **Nucleosome** destabilizing effects were observed with many DNA-intercalating compounds [17, 20, 21], as well as with some compounds binding within the DNA minor groove [22]. However, these studies went largely unnoticed by the cancer community, which traditionally attributed the cytotoxic and anti-cancer activity of DNA-binding compounds to their ability to induce DNA damage or breakage. For example, it was shown that while etoposide and doxorubicin are both categorized as DNA damaging drugs, they have different therapeutic effects. Etoposide is not efficient as a single agent, whereas doxorubicin monotherapy is the standard of care for the treatment of soft tissue sarcomas and many other tumors including breast and ovarian cancer and many leukemias [23–25]. The reason for the clinical superiority of doxorubicin monotherapy was unknown until it was noticed that doxorubicin causes histone eviction from chromatin in cancer cells [15, 26].

The anti-cancer activity of anthracyclines, the group of drugs to which doxorubicin belongs, is attributed to multiple mechanisms, including poisoning of topoisomerase II, inhibition of DNA replication and transcription, and induction of reactive oxygen species (ROS) [27]. However, the major reason for cancer cell death stemming from all these mechanisms was believed to be DNA damage until a variant anthracycline, aclarubicin (Figure 1), was identified as an effective anti-cancer drug in treatment of patients with acute myeloid leukemia (**AML**) [28, 29]. In contrast to doxorubicin, aclarubicin causes no DNA damage while still inducing histone eviction [13–16, 26].

After these discoveries, the difference between etoposide and doxorubicin efficacy was revisited. Etoposide was shown to induce DNA breaks at topoisomerase II sites including the well-characterized site at 11q23 underlying treatment-related KMT2A-rearranged acute leukemias [30]. Specifically, etoposide binds to the DNA-topoisomerase complex when DNA is already cleaved by the topoisomerase and inhibits DNA ligation leading to DNA breaks [31]. On the other hand, anthracyclines inhibit the nucleolytic activity of topoisomerase at therapeutic concentrations, preventing repair of DNA breaks formed by topoisomerase II in the cycle of DNA break, DNA unwinding and DNA repair, as critical for -for example- efficient transcription [32]. DNA damaging activity of some anthracyclines stems from their ability to cause metal ion dependent DNA oxidation [33]. However, in addition, doxorubicin evicts numerous histones, including H2AX. Phosphorylated H2AX is an important marker for the DNA damage response, which leads to DNA repair [34]. Eviction of H2AX then induces considerably slower DNA repair in cells exposed to doxorubicin unlike cells exposed to etoposide that does not evict H2AX [26]. It is likely that the slowed DNA repair after doxorubicin treatment induces more efficient cell death but also allows accumulation of mutations, increasing chances of development of secondary tumors [13].

Comparison of different anthracyclines showed that histone evicting-activity correlates best with anti-neoplastic effects, whereas cardiotoxicity, infertility and secondary cancers are associated with DNA damage [13, 14, 35]. These correlations are best illustrated by the anthracycline variant aclarubicin that only evicts histones and then lacks the ability to induce cardiotoxicity and form secondary cancers when compared to the classical genotoxic anthracyclines [13, 36, 37]. Variations can easily be made within the chemical space of anthracyclines to generate variants that have lost their DNA damage activities while maintaining histone eviction activity. Surprisingly, these ‘histone eviction only’ variants have not lost any cytotoxic activity unlike the variants that only are able to induce DNA breaks, like amrubicin, which is a relatively poor cytotoxic anthracycline. These data suggest that histone eviction is the major cytotoxic activity in the classical anthracyclines used in the clinic [13, 14, 35, 38].

Similar to some anthracyclines, another group of DNA-intercalating compounds, **curaxins** (Figure 1), lack DNA-damaging activity but have anti-neoplastic effects [39]. These compounds were discovered in a screen for p53 activation in tumor cells, where DNA-damaging compounds did not activate p53 [40]. Curaxins bind to DNA via insertion of a carbazole moiety between base pairs and side chains into minor and major grooves of DNA. Curaxins have demonstrated anti-cancer activity in multiple preclinical cancer models [39, 41–45]. Their cytotoxicity to tumor cells has also been correlated to their ability to destabilize nucleosomes and to cause histone eviction [39, 46, 47]. At the same time, their DNA-damaging activity was undetectable in mammalian cells [39, 46, 48]. Like aclarubicin, curaxin CBL0137, acting only by histone eviction, does not increase incidence of therapy induced tumors and infertility in mice when compared to doxorubicin [39, 44]. 

Collectively, the data obtained with anthracycline variants and curaxins suggest that chromatin destabilization may be the major cytotoxic activity when compared to DNA damage. This ‘new activity’ present in old cancer drugs provides new opportunities for further drug development. Since different anthracyclines target different genomic areas for histone eviction [36, 49], this may have different effects on specific tumors with a different (epi)genetic makeup.

## Distinguishing DNA damage from chromatin damage  

DNA damage (Box 1) is defined as any chemical modification of DNA resulting in the breaking of existing covalent bonds (*e.g.*, DNA breaks) or forming new ones (e.g., platinum adducts), whereas chromatin damage (Box 2) refers to loss of histones from chromatin due to the change of biophysical properties of DNA (e.g., length, charge, twist, and flexibility) and disruption of histone - DNA interactions. For some molecules, DNA and chromatin damage are almost inseparable (Figure 1). For example, cis-platinum compounds, which bind covalently to the same DNA strand, cause a bend of DNA helix, leading to chromatin damage [47, 50]. Trans-platinum compounds, which bind to opposite DNA strands and cause similar DNA damage but no bending, are very poor anti-cancer agents [51]. However, for many other compounds, there is no direct connection between DNA and chromatin damage (Figure 1). In an attempt to separate DNA damaging activity of anthracyclines from their chromatin damaging activity, the chemical space of anthracyclines was explored, and many variants were made and tested. Comparison of anti-cancer activity of these variants demonstrated that chromatin damage rather than DNA damage is the dominant anti-cancer activity, as illustrated by aclarubicin, an anthracycline variant that acts exclusively by inducing chromatin damage and is an effective drug for patients with AML [14, 15, 26, 35, 36, 52]. DNA-damaging compounds in the anthracycline group probably cause DNA damage via induction of ROS due to the conversion of quinone to semiquinone by a number of reductases in cells [53, 54]. However, the doxorubicin analogue amrubicin is poorly cytotoxic, despite inducing ROS formation [55]. Thus, release of histones along with other chromatin associated proteins from the genome may be a dominant cytotoxic mechanism used by anthracycline and curaxin cancer drugs.

## Chromatin damaging compounds differ from other epigenetic therapies

The common property of intercalators sets chromatin damaging drugs apart from other epigenetic drugs, which act by directly inhibiting chromatin modulators, such as chromatin **“writers”**, e.g., **DNA methyltransferases**, **histone deacetylases** (HDACs) and **“readers**”, e.g., bromodomain proteins. Chromatin damage induced by intercalators is a spontaneous process and does not involve specific enzymes. Consequently, mutations in critical enzymes may not affect the chromatin damaging activity of anthracyclines or curaxins. However, different degree of pre-existing chromatin destabilization or the level of expression of **histone chaperones** may influence the sensitivity of cells to chromatin damaging therapy. Since anthracyclines have different chromatin specificities [36, 49], different drugs may affect tumors with a different chromatin state. Consistent with these mechanistic differences, the HDAC inhibitors, such as valproic acid, tucidinostat or panobinostat, synergize with doxorubicin and curaxins by making DNA more accessible and changing the chromatin state for histone eviction [56–60]. This possible synergy may lead to more effective cancer therapies.

While chromatin damage by anthracyclines and curaxins has a global impact on genomic structures [61, 62], including those not marked by specific epigenetic marks [36], epigenetic drugs affect only specific modifications. In addition, altering epigenetic marks often requires long drug exposure as these modifications are relatively stable. On the other hand, chromatin damaging drugs work very swiftly in evicting histones (in a matter of minutes) [26, 47]. This implies that first using chromatin damaging drugs to release the epigenetically marked histones followed by a maintenance treatment with epigenetic drugs could be an interesting combination treatment for swift epigenetic control in regions defined by the anthracyclines used.

## Cellular consequences of chromatin damage

**Figure 2.**
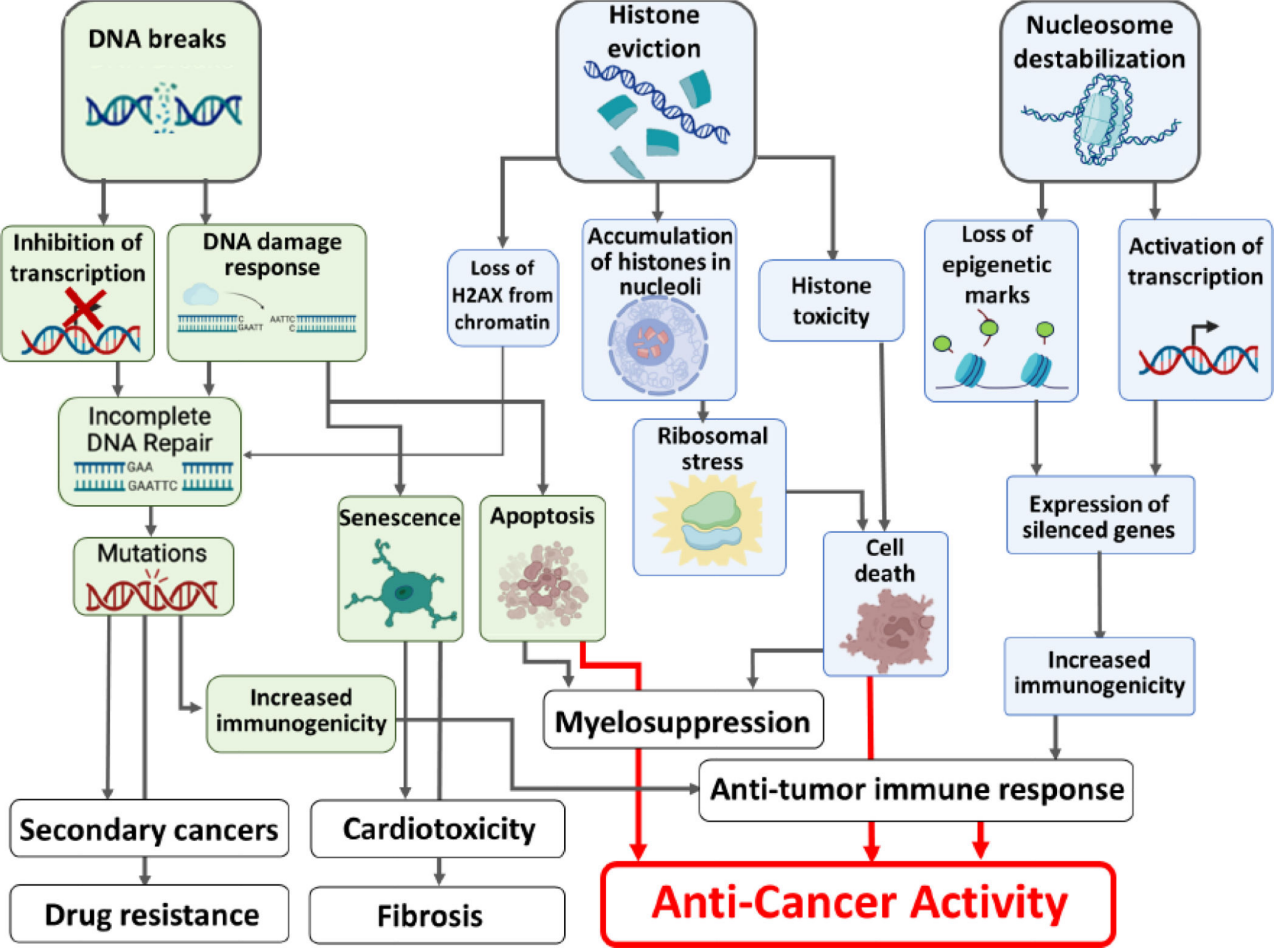
Cellular consequences of DNA and chromatin damage (green and blue shapes respectively, see also Box 3) and their clinical manifestations (white shapes). Created with BioRender.com

The immediate and specific effect of chromatin damage is the destabilization and disassembly of nucleosomes and release of histone proteins into non-chromatin nuclear compartments, e.g., in nucleoli (Box 3) and cytoplasm [63] (Figure 2).  Both anthracyclines and curaxins also disrupt 3D genome organization [61, 62, 64]. Besides effects on nucleosomes, these drugs disrupt the DNA binding of other proteins, including HMGB1, linker histones, transcription factors and CTCF [61, 65, 66]. The chromatin binding protein landscape is thus altered by these anti-cancer drugs. 

Changes in nucleosome occupancy may have a very strong effect on the binding of chromatin-associated factors. The histone chaperone FACT [39, 47] (Box 3) is an important example. FACT binds histone oligomers through different domains of two subunits, SSRP1 and SPT16. However, FACT binding sites are not exposed when the histone core is wrapped with DNA. This leads to FACT binding to chromatin at the regions of high transcription, where nucleosomes are frequently unwrapped due to passage of RNA polymerases [67]. In case of chromatin damage, nucleosomes become unwrapped in different genomic regions leading to FACT binding away from sites of transcription, referred to as chromatin trapping or **c-trapping** of FACT (Box 3) [47]. Intercalators can also affect binding of topoisomerases to DNA, and these can be trapped on chromatin by the anthracycline inhibitors [14, 16, 66, 68].

Histones are positively charged proteins capable of non-specific binding to any nucleic acid, including RNA with an affinity 100 times higher than DNA [69]. When histones are mixed with DNA at physiological salt concentration, they do not form nucleosomes, but irregular nucleo-protein precipitates.  In non-chromatin-damaging conditions, such as during replication or DNA repair, non-chromatin histones are bound by histone chaperones [70]. Histone chaperones shield the positive charge of histones allowing their proper assembly before exposure to DNA, thus preventing irregular binding to nucleic acids. Evicted histones can be transported into the cytoplasm to interact with mitochondria for apoptosis induction [63]. Although the consequences of free histone accumulation in cells are poorly studied, microinjection of free histones was shown to result in apoptosis [71] (Box 3), suggesting that histone release following chromatin damaging drug treatment may be a trigger for cell death.

## Chromatin damage and transcription 

Chromatin damaging compounds activate transcription at lower concentrations and generally inhibit transcription at higher, often toxic, concentrations [48]. Increased transcription is exemplified by p53 targets or interferon responsive genes [39, 72] (Box 3). It is unclear how chromatin damage activates the signaling pathways controlling these genes or alternatively transcription of these genes are activated due to the disassembly of nucleosomes at the promoters of these genes allowing access by the transcription machinery [48].

The effect of chromatin damaging drugs on transcription can be interpreted in terms of the twin-supercoiling domain model [73]. When an RNA polymerase is engaged in transcription, it pushes forward a denaturation bubble, which results in a wave of positive torsion (over-winding of the double helix) ahead and a wave of negative torsion behind. Topoisomerases sense and relieve the torsion by transient cleavage and ligation reactions to prevent stalling that would otherwise inhibit transcription [74]. The DNA that wraps around a nucleosome is negatively supercoiled. Consequently, the positive torsion ahead of an RNA polymerase forces nucleosome unwrapping. DNA intercalators, by changing the nucleosome-constrained supercoiling of DNA, could facilitate its unwinding from the nucleosome and enhance transcription. Alternatively, if intercalators stabilize the cleavage complexes of both topoisomerase I and II, they may impede RNA polymerase transit.

In contrast to most anthracyclines, DNA intercalation by aclarubicin does not cause DNA damage [16], but it similarly alters transcription [15, 26]. Aclarubicin treatment may evict histones in genes to stimulate RNA Polymerase II (RNAPII) elongation. In addition, this may increase chromatin accessibility at promoters, especially at closely spaced divergent ones, where increased negative supercoiling concentrated between diverging RNAPIIs could increase drug binding to accumulate chromatin damage [75]. As the transcriptional effects of detoxified doxorubicin [13] and other chromatin-damaging intercalating drugs are investigated, it will be interesting to determine whether they have similar torsion-mediated effects on genes transcribed by different RNA polymerases. 

## Cytotoxicity of chromatin damage

Cytotoxicity caused by chromatin damage may stem from several non-exclusive mechanisms, including transcriptional dysregulation, toxicity of free histones, disruption of ribosomes caused by nucleolar histone accumulation, loss of function of chromatin regulators, such as FACT, or activation of stress-related pathways, such as p53 or interferon type I (Figure 2 and Box 3) [39, 72]. Although the last two factors may play a role in cytotoxicity, they are not major factors, since cells with disabled p53 or interferon receptor still die from chromatin-damaging agents, though at higher drug concentrations [39, 72].

An important distinction between cytotoxic effects of chromatin-damaging agents from DNA-damaging agents is the absence of senescence, as observed for curaxins [48]. Cells either die from curaxins or resume proliferation upon compound removal. The latter may appear disadvantageous for cancer treatment. However, induction of senescence may also be deleterious for tumor treatment, since senescent cells are resistant to cell death and produce pro-inflammatory factors manipulating the tumor microenvironment into a more drug resistant state.

## Activation of anti-tumor immune responses by chromatin damage

The efficacy of curaxin CBL0137, as well as anthracyclines is stronger in immune-competent than immune-deficient mice [76, 77]. This is surprising as both agents are lymphodepleting and myelosuppressive (Figure 2). However, these drugs can induce type I interferon responses in tumor cells, which makes them visible to the immune system [72, 78] despite concurrent inhibition of the pro-inflammatory factor NF-kB [39]. This could be a basis for a phenomenon called chemo-immunotherapy where chemotherapy strongly boosts anti-cancer immune responses. Anthracycline treatment can activate cGAS-STING detection of cytoplasmic DNA emerging from DNA breaks caused by topoisomerase inhibition [77, 78]. However, CBL0137 does not cause DNA breaks but rather chromatin damage following histone eviction, leading to exposed DNA ends that could support cGAS-STING activation. Other mechanisms of immune system activation may include formation of **Z-DNA** [47, 79] or activation of MHC genes, as well as inactivation of immunosuppressive factors [76]. In addition, chromatin damage may change the transcription program in immune cells, such as decondense chromatin in regulatory T cells (Tregs) and in exhausted T-cells and thus change their phenotype from immune suppressive to pro-active. Tumor cells may also die through a phenomenon called immunogenic cell death that then boost immune responses [80]. In summary, anti-cancer drugs, such as curaxins and anthracyclines, have multiple effects on the immune system to further detect and eliminate cancer cells.

## Why are tumor cells more sensitive to chromatin damage than normal cells?

We propose that tumor cells are more vulnerable to chromatin damaging therapy due to their more dynamic and less stable chromatin state. This is also suggested from dose-response experiments on cell lines with DNA damage or histone evicting anthracycline variants [13, 36]. The question is then how chromatin damage provides a therapeutic window for cancer treatment. This is unclear. It was shown that nucleosomes become generally more dynamic during oncogenic transformation [81], which may make transformed and tumor cells more sensitive to chromatin damaging drugs [48]. However, chromatin damaging drugs, like aclarubicin, can also affect normal tissue, especially white blood cells [82]. On the other hand, while nucleosome instability is emerging as a novel cancer driver [83–90], massive nucleosome destabilization is lethal in eukaryotes [91, 92].

Nucleosome stability defines DNA access of the transcriptional machinery. Tumor cells are characterized by phenotypic plasticity [93], which requires easy and fast transition between different transcriptional programs. Normally, these transitions are limited by chromatin organization, including stable nucleosomes at the promoters of silenced genes and location of silenced genomic regions in non-transcribed nuclear territories. Destruction of chromatin organization in tumor cells may be achieved if nucleosome stability is reduced genome-wide, such that all chromatin in cells is less condensed. This does not only make access of transcription factors to DNA easier, but also enables chromatin fibers to be more flexible and mobile, which would facilitate promoter-enhancer contacts and switches of transcriptional programs [83]. Consequently, if chromatin in tumor cells undergoes higher histone turnover, then lower concentrations of chromatin damaging drugs would be required for losing histones from chromatin and initiate cell death. Indeed, anthracyclines bind better to open chromatin [26, 36, 49]. The chromatin state is then important for allowing histone eviction by chromatin damaging cancer drugs. Different anthracyclines target different genomic regions for histone eviction [36] and may then be better for tumors with a defined chromatin state. If so, this would open the doors of chromatin defined personalized therapy with anthracycline drugs that have already been shown to be effective across many tumor types.

## Concluding remarks

To realize the full potential of chromatin-damaging therapy, as well as associated risks, better understanding of biology and chemistry of chromatin-damaging compounds is needed (see Outstanding Questions).

Currently clinical experience with chromatin damaging drugs is limited. Aclarubicin was first used in patients in the late 1970s in Phase I trials for refractory liquid and solid malignancies. Interest stemmed from two findings in pre-clinical models: reduced cardiotoxicity and increased uptake in cancer cells resistant to doxo- and daunorubicin via overexpression of Multidrug Resistance 1 (MDR1) [28, 29]. Phase I/II trials in heavily pre-treated patients with acute myeloid and lymphoid leukemias showed encouraging results. In one study, 11/25 AML patients (44%) achieved complete remission. No cases of depressed cardiac function were identified, despite inclusion of patients ranging from age 2 to 80 years [37, 94, 95]. Aclarubicin was also used in place of daunorubicin in a Phase I pediatric AML trial and showed similar response rate as daunorubicin (80.0 vs 82.2%) [96, 97]. On the other hand, when aclarubicin was tested as monotherapy in breast and lung cancer, no significant clinical response was observed, perhaps due to poor distribution in those tissues [98, 99]. Taken together, these results illustrate that the class of anthracyclines should be divided to a series of different drugs that should be optimized for specific tumor types.

Curaxin CBL0137 has been tested in Phase I dose escalation studies in patients with relapsed and refractory solid tumors, including breast cancers, sarcomas, liver cancer and GU malignancies (NCT01905228). No complete responses were observed, though several patients had stable disease or partial responses. Side effect profiles were quite manageable and included photosensitivity and thrombocytopenia. Because of the favorable side effect profile, CBL0137 could be administered for as long as 24 months. CBL0137 is now being tested in trials for children and young adults with solid tumors and CNS malignancies (NCT04870944), as monotherapy in melanomas and sarcomas (NCT03727789), and in combination with ipilimumab and nivolumab in melanoma (NCT05498792). To define the prospects of chromatin damage therapy, clinical trials are needed to compare efficacy and safety of compounds causing only DNA damage, both DNA and chromatin damage and only chromatin damage. The activity of aclarubicin-based therapies in the treatment of patients with AML already indicated the clinical application of chromatin-damaging therapy. In addition, side effects of the currently used anthracyclines, such as infertility and treatment-induced secondary tumors are usually not considered a major issue or simply not followed by the treating oncologists. Yet, they may be controlled by anthracycline or curaxin variants that only employ chromatin damage as anti-cancer activity. Introducing such drugs will also improve quality of life and decrease health costs for cancer survivors later in life. Chromatin damage as a concept in drug development provides a new opportunity to develop less toxic and more efficacious therapies that are poised to make cancer treatment more accessible, equitable, and applicable to all patients, regardless of age, disease, or comorbidity status.

## Box 1. DNA binding and DNA damage

Small molecules can form covalent or non-covalent bonds with DNA. Covalent DNA binders cause DNA damage following chemical modification of DNA (platinum compounds and alkylating agents). Non-covalent molecules bind DNA via hydrogen bonding, Van der Waals forces, π–π interactions, etc. These compounds do not cause direct DNA damage. Non-covalent binding of small molecules to DNA may occur via intercalation between base pairs, or binding to minor or major grooves of DNA helix.

Covalent and non-covalent bulky compounds interfere with the activity of enzymes that use DNA as a substrate, e.g., DNA polymerases, which may lead to replication fork collapse and single- or double-strand breaks. A special case of these enzymes is **topoisomerases**. These are among the first established and most widely used targets of anti-cancer agents. Importantly, these enzymes perform at least two catalytic activities: they cut DNA (single or double strand) and then ligate. DNA breaks from **podophyllotoxins** or **camptothecins** are results of inhibition of topoisomerase ligation. These compounds do not bind to naked DNA but only those that are cleaved and bound by topoisomerases.

Anthracyclines are another class of topoisomerase inhibitors, which intercalate into naked DNA. There are still uncertainties about the mechanism of topoisomerase inhibition by these compounds since there is no crystal structure of a DNA/topoisomerase/anthracycline complex. At therapeutically relevant concentration of anthracyclines, topoisomerase II cannot cleave DNA. This mode of inhibition does not lead to DNA breaks. The same intercalation mode of topoisomerase inhibition is found for curaxins. However, some anthracyclines, such as doxorubicin, cause DNA breaks in cells. The most likely reason is direct chemical reaction with DNA due to the presence of specific chemical moieties, some of which were recently identified [35]. Other anthracyclines lack reactive moieties (aclarubicin) and bind DNA and inhibit topoisomerase II without causing DNA damage.

## Box 2. Chromatin and chromatin damage

Chromatin is a nucleoprotein complex. The nucleosome is a unit of chromatin, consisting of histone core, four pairs of core histone proteins H2A, H2B, H3 and H4, wrapped with 147 bp of DNA ~1.7 times. The histone core may be viewed as a disk with centrally located tetramer of two H3 and two H4 histones and two dimers of H2A and H2B attached from the front and back of the tetramer. **Nucleosomal DNA** wraps the disk starting from an H2A/H2B dimer, then covering the ridge of tetramer and exiting via the second H2A/H2B dimer. Nucleosomes are connected by 40–60 bp of **linker DNA**, which may be locked down with linker histone H1 binding to the DNA at entry and exit sites and the core histones. Histone proteins have multiple variants and post-translational modifications, some of which transmit instructions to transcriptional regulatory machineries as to which regions may or may not be made accessible for processes, such as transcription and DNA repair.

The binding of small molecules to DNA alters the stability of nucleosomes via different mechanisms: stretching of DNA, untwisting the helix, reducing DNA negative charge (most of DNA ligands are positively charged) and altering DNA flexibility. This leads to DNA unwrapping from the core, making histones prone to loss from chromatin. We refer to this phenomenon as histone eviction from chromatin, and accumulation of free histones in non-chromatin cell compartments as chromatin damage. In some physiological situations, histones are removed by chromatin remodelers and in this case, they are “accompanied” by histone chaperones, which mitigates the consequences of chromatin damage.

The relationship between DNA damaging and chromatin damaging activities of small molecules has not been firmly established. We propose that these two activity modes can be fully separated for some types of molecules at least in their short-term effects. However, in the long-term, DNA damage may accompany disassembly of chromatin to provide access to the DNA repair machinery. Naked DNA is more exposed to water molecules, which are source of ROS and therefore “secondary” DNA damage is sometimes observed upon treatment of cells with “pure” chromatin damaging agents. However, emergence of significant amounts of naked DNA following chromatin damage leads to cell death and it is hard to discern the exact mechanisms of DNA damage in a dying cell. Chromatin damage may also remove the histone variant H2AX that is phosphorylated for DNA repair. For instance, drugs such as doxorubicin that combine chromatin damage with DNA damage result in slower DNA repair.

## Box 3. Cellular consequences of chromatin damage

The following phenomena are observed in cells upon treatment with chromatin damaging compounds. Although some of them have similarities with the effects of DNA damage, they have features specific to chromatin damage. Mechanisms of some of these effects are not fully understood. However, they are important for the short- and long-term consequences of chromatin damage.

- **Chromatin trapping (c-trapping)** of FACT. FACT does not bind assembled nucleosomes that are fully wrapped with DNA since FACT-binding epitopes are hidden. When DNA is unwrapped from histone core, these epitopes become exposed and available for FACT binding. FACT can also bind DNA in a non-sequence-dependent manner. DNA unwrapping from a nucleosome caused by chromatin damaging agents first result in exposure of histone epitopes, which are bound by different domains of FACT [47]. When DNA is significantly unwrapped, it accumulates negative supercoils and can transit to a left-handed DNA helix, known as **Z-DNA**. FACT binds Z-DNA via the c-terminal domain of its SSRP1 subunit.
- **Nucleolar Histone Accumulation (NuHA)** is observed in the case of histone expression from a strong exogenous promoter or chromatin damage. All four core histones and linker histone H1 can accumulate in nucleoli in curaxin and aclarubicin treated cells [46, 47]. Interestingly, NuHa is not observed in doxorubicin treated cells, although some histones are detected in the cytoplasm. Whether nucleoli serve as buffers for extra histones, or this accommodation just disrupts nucleoli function leading to “ribosomal stress” is unclear.
- **Activation of p53** in response to chromatin damage occurs via accumulation of p53 protein, similar to the DNA damage response, but in the absence of post-translational modifications characteristic to DNA damage. There is also no detectable activation of upstream kinases, such as ATM, ATR, CHK1 and CHK2 [39, 48]. After chromatin damage, p53 accumulates in higher levels than after DNA damage, but its transcriptional activity is comparable to its activity after DNA damage, probably due to lower activity of the unmodified protein [48]. One possible mechanism of p53 activation is phosphorylation of serine 392 by casein kinase 2 in complex with FACT [100], and another may be related to NuHA: histones accumulated in nucleoli may disrupt nucleolar chaperones binding to inhibitors leading to their eviction from nucleoli and binding to MDM2, allowing p53 accumulation.  
- **Activation of the type I interferon sensitive genes** is a major transcriptional response to curaxin CBL0137 in cells, mice, and humans [72]. Potential mechanisms may include accumulation of dsRNA, most probably due to divergent transcription from pericentromeric and centromeric regions becoming accessible to the transcription machinery due to chromatin decondensation.

## Outstanding questions

- How does chromatin damage affect the epigenetic state of tumor cells?
- Does the chromatin state of tumors confer selectivity to chromatin damaging drugs?
- How is chromatin selectivity of anthracycline drugs defined? And how does this relate to tumor targeting of these drugs?
- How does chromatin damage translate into cell death?
- How can the optimal anthracycline variant be selected given the genomic status of a tumor type?
- Can chromatin-damaging drugs be improved?
- Does the eviction of non-histone chromatin proteins other than histones contribute to chromatin damage?

## Highlights

- Chromatin modifiers are among the most commonly mutated classes of genes in cancer.
- Chromatin-damaging activities disrupt histone-DNA interactions without damaging DNA.
- Chromatin damage may explain the efficacy of anthracyclines in a broad range of liquid and solid cancers.
- The chromatin-damaging anthracyclines tested in animals, or in the clinic retain anti-neoplastic efficacy of DNA-damaging drugs without irreversible side effects.
- Understanding the molecular consequences of chromatin damage will maximize the impact of chromatin damaging drugs in oncology.

## Glossary

AML: acute myelocytic leukemia, type of hematologic malignancy
Anthracyclines: a group of antibiotics produced by certain types of Streptomyces bacteria as well as their half-synthetic variants. They poison topoisomerase II and are one of the major types of chemotherapeutic anti-cancer agents. Examples are doxorubicin, daunorubicin and aclarubicin
Camptothecins: group of chemotherapy drugs structurally similar to campothecin, inhibitors of topoisomerase I. Examples are campothecin, irinotecan, and topotecan
Chemotherapy: cancer treatment utilizing drugs that do not target tumor specific factors, but rather factors important for cell division in normal and tumor cells, in contrast to targeted therapy where drugs inhibit factors more selective for tumor tissue
Chromatin: a nucleoprotein complex of DNA,histone and non-histone proteins, a form of organization of genomic DNA in eukaryotes
Curaxins: a group of structurally similar carbazole-based experimental anti-cancer agents. An example is CBL0137
DNA methyltransferases: enzymes that add a methyl group to the cytosine base of DNA
Genotoxicity: ability to cause genetic mutations
Histone acetyl transferases (HAT): enzymes that add acetyl groups to histones
Histone chaperones: nuclear factors that bind histone proteins and facilitate their assembly into nucleosomes
Histone deacetylases (HDAC): enzymes that remove acetyl groups from histones
Linker DNA: DNA between neighboring nucleosomes
Nucleosomal DNA: DNA helix that wraps a nucleosome core
Podophyllotoxins: a group of chemotherapeutic anti-cancer agents, inhibitors of topoisomerase. Examples are etoposide and teniposide
“Readers”: proteins that recognize and bind histone posttranslational modifications
Secondary cancers: tumors emerging in patients as the result of an earlier treatment of unrelated (primary) cancer
Topoisomerases: enzymes that relieve DNA helix over- or under-twisting as well as knotted DNA helices by catalyzing DNA cleavage, relaxation and relegation, thus permitting changes in DNA topology
“Writers”: enzymes that add posttranslational modifications to histones such as methylation, acetylation, ubiquitination
Z-DNA: DNA in the form of a left-handed helix, opposite to the right-handed B-DNA in physiological conditions
